# Herbivory by *Atta vollenweideri*: Reviewing the significance of grass-cutting ants as a pest of livestock

**DOI:** 10.3389/finsc.2023.1101445

**Published:** 2023-04-05

**Authors:** Julian Sabattini, Martin Bollazzi

**Affiliations:** ^1^ Consejo Nacional de Investigaciones Científicas y Técnicas, Cátedra de Ecología de los Sistemas Agropecuarios, Facultad de Ciencias Agropecuarias, Universidad Nacional de Entre Ríos, Oro Verde, Argentina; ^2^ Entomología, Facultad de Agronomía, Universidad de la República, Montevideo, Uruguay

**Keywords:** leaf-cutting ants, pests, impact, livestock, rangelands, foraging

## Abstract

The grass-cutting ant *Atta vollenweideri* is well suited for studies examining the negative effect leaf-cutting ants have on livestock production in South American grasslands because they forage on the same plants as cattle. This study investigated the impact of *A. vollenweideri* on livestock production in Argentinean rangelands. First, we assessed *A. vollenweideri* herbivory rates and its economic injury level (EIL). Second, using satellite imagery in a region covering 15,000 ha, we estimated the percentage of this area that surpassed the calculated EIL. Results showed that *A. vollenweideri* consumed approximately 276 kg of dry plant weight/ha/year, foraging mostly on grasses (70%). Additionally, ants cut 25% of herbs and 5% of trees. In summer and autumn, ants consumed more grasses, while in winter and spring, herbs and trees were also significantly cut. Ants consumed 7% of the forage demand needed to raise a calf according to the management regime applied by farmers. Our calculated EIL (5.85 nests/ha) falls in the range of previous studies. Colonies were absent in 93.6% of the surveyed area, while their density was below the EIL in 6.2% of the area. *A. vollenweideri* populations surpassed the EIL in only 0.2% of the area, which corresponds to 2.6% of the locations holding colonies. These results question the perception that *Atta* leaf-cutting ants are a pest of livestock production. Although ants consume a small percentage of cattle’s forage demand, evidence that ants and cattle are competing in the few cases in which density surpasses the EIL is arguable. First, grass-cutting ants are capable of consuming herbs and trees in addition to the grasses on which cattle mostly feed. Second, there is no evidence indicating that both are cutting the same plant portions when preferences overlap. Third, evidence suggests that ants are not displaced under high-pressure grazing regimes by cattle. In the countries where *A. vollenweideri* is present, decision makers have promulgated several acts making its control mandatory. It is time to revisit the pest status of *A. vollenweideri* and include the use of EIL as a control criterion.

## 1 Introduction

Leaf-cutting ants belonging to the genera *Atta* and *Acromyrmex* are primary pests and have been considered as the insects that cause the most damage to agriculture throughout the Neotropics (1). During foraging, leaf-cutting ant workers cut and transport plant fragments, which are then used to cultivate a symbiotic fungus inside the so-called fungus chambers (2). This fungus serves as the sole food source for the colony (3). *Atta vollenweideri* (Hymenoptera: Formicidae) is well suited for studies examining the negative effect leaf-cutter ants may have on extensive livestock practices in South American grasslands. They belong to the group classified as grass-cutters together with *Atta capiguara* and *Atta bisphaerica* (4, 5). Grass-cutting ants have traditionally been seen as the main cattle competitor because they forage on the functional group on which cattle mostly feed (6). *A. vollenweideri* does not show homogenous distribution on a local scale because it tends to nest on heavy clay soils used for livestock production due to its low productivity for cropping (7–9). **Table 1** summarizes a comprehensive list of studies reporting plant consumption over the last decades. To date, three publications have analyzed consumption by *A. vollenweideri* and its possible impacts on livestock. While the study carried out by Robinson and Fowler (13) reported a consumption of 868 and 924 kg of dry weight/ha/year, Jonkman (12) estimated a consumption of only 38 kg of dry weight/ha/year. Based on those results, Robinson and Fowler (13) concluded that *A. vollenweideri* consumed twice the yearly requirement of cows. However, Jonkman (12) estimated it was below 5%. Both studies, nevertheless, suffer from having estimated the consumption based on only one *A. vollenweideri* nest. Additionally, they did not cite the aerial net primary productivity (ANPP) of the habitat, which determines the amount of net available forage source for which ants and cows should compete. The most comprehensive study was conducted by Guillade and Folgarait, who also measured ANPP and the portion consumed by cattle (14). They reported a minimum of 224 and a maximum of 1,867 kg of dry weight/ha/year, averaging 1,216 kg of dry weight/ha/year consumed by *Atta vollenweideri*. The authors concluded that competition between ants and cattle occurred only during the low-productivity periods of the year. The economic injury level for the study location was estimated at 0.29 nest/ha.

**Table 1:** Average intake by colonies of *Atta* species obtained from the literature, as well as the method applied and the number of colonies assessed.

Species	Location	Reference	Colonies	Method	Intake (kg/ha/year)
*Atta capiguara*	Brazil	Amante 1972 (10)	?	Foraging activity	1,015
*Atta capiguara*	Brazil	Forti 1985 (11)	?	Foraging activity	165
*Atta vollenweideri*	Paraguay	Jonkman 1980 (12)	1	Conversion	36
*Atta vollenweideri*	Paraguay	Robinson and Fowler 1982 (13)	1	Foraging activity	868
*Atta vollenweideri*	Paraguay	Robinson and Fowler 1982 (13)	1	Exclusion	924
*Atta vollenweideri*	Argentina	Guillade and Folgarait 2015 (14)	3	Exclusion	1,216
*Atta cephalotes*	Costa Rica	Blanton and Ewel 1985 (15)	4	Foraging activity	653
*Atta colombica*	Costa Rica	Lugo et al., 1973 (16)	1	Foraging activity	312
*Atta colombica*	Panama	Herz et al., 2007 (17)	50	Foraging activity	132.4
*Atta colombica*	Panama	Haines 1978 (18)	9	Conversion	98
*Atta colombica*	Panama	Wirth et al., 1997 (19)	2	Foraging activity	517
*Atta opaciceps*	Brazil	Siqueira et al., 2018 (20)	8	Foraging activity	597
*Atta opaciceps*	Brazil	Costa et al., 2008 (21)	8	Foraging activity	824

Only studies that provided detailed information on methods were taken into account, resulting in data for two grass-cutter ants (*A. vollenweideri* and *A. capiguara*) and three leaf-cutter ants (*A. colombica*, *A. cephalotes*, and *A. opaciceps*). The revision of data from Amante 1972 (10) conducted by Fowler, Forti, and Romagnano (22) are given instead of the values provided by the author (ca. 6,259 dry weight in kg/ha/year). The data provided by Robinson and Fowler (13) for *Atta capiguara* were not included because the authors considered them as inconclusive. A full description and discussion of methods can be found in Fowler et al. (22) and Guillade and Folgarait (14).

In addition to estimating consumption by ants and the pasture demands of cattle, a comprehensive estimation of competition between leaf-cutting ants and cattle should also include an estimation of nest density at a landscape scale, and not only the density of the study site where measurements have been performed. Leaf-cutting ant distribution is not homogeneous and tends to be aggregated at both local and landscape scales (7, 23, 24). When actual density at landscape scales is not determined, and the impact of leaf-cutting ants on cattle is extrapolated from the highly infested areas where consumption was measured, the negative effect of ants on livestock can be overestimated. An extrapolation to landscape level should also be carried out to properly discuss the impact of leaf-cutting ants.

Thus, this study aimed to study the potential impact of *A. vollenweideri* as a pest at both local and landscape scales. Locally, we assessed functional groups foraged by *A. vollenweideri* (grasses, shrubs, and trees) to estimate the potential impact of ants by considering the extent to which ant preferences might overlap with the known grass preference of cattle. We also calculated the impact of such overlapped consumption by considering the standard cattle management regime for the region. Second, working at a regional scale in midwestern Argentina, we estimated nest density through satellite images. This was carried out to assess the percentage of the area that surpass the critical density at which *A. vollenweideri* may negatively affect production.

## 2 Methods

### 2.1 Plant consumption by colonies

At a local scale, methods were, first, aimed at assessing seasonal herbivory rates by the leaf-cutting ant *A. vollenweideri* using foraging activity methods, while considering plant functional groups. Second, we compared ant consumption with the reported forage demands of cattle and the ANPP.

**Figure 1:**
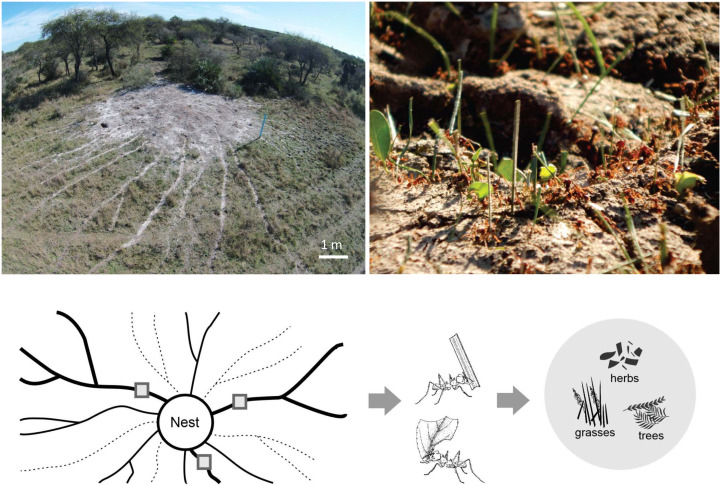
Top left: picture of an *Atta vollenweideri* nest taken by a drone at the El Caraya study site. Foraging trails can be clearly seen departing radially from the nest mound. Top right: picture showing the loads carried by *A. vollenweideri* workers entering the nest. Most loads belong to grass fragments but some of them are pieces cut from herbs and trees. See also **ESM 1** for a video showing typical foraging activity in a trail. Bottom: diagram showing the methods for estimating plant consumption. At the day of measurement, foraging trails can be active (represented by continuous lines) or inactive (represented by dashed lines). Among the actives, three equidistant trails were selected (thick trails). In these trails, three collecting points were marked (grey quadrants). Three people working simultaneously collected all workers and their loads in a 5 min period every 2 h over 24 h. Loads were later classified into grasses, herbs, and trees for obtaining daily and seasonal intakes. See Methods for further details.

We selected two sites in the province of Entre Rios, Argentina, with high-density *Atta vollenweideri* populations. “El Caraya” (-30.633356, -58.847075) has a nest density of 1.27 nest/ha, while “Santa Clara” (-31.549827, -59.677187) has a nest density of 1.63 nest/ha. Livestock have been historically bred at both sites and there were no records of agriculture or pasture improvement. Both sites possess the representative soil type Vertic Epiaqualfs (8) and were located approximately 130 km from each other. In each site, five mature nests located far from any perturbation (i.e., roads and houses) were selected (**Figure 1**). Those nests had an average diameter of 7.8 m (± sd 1.1, n=10), a height of 0.45 m (± sd 0.16, n=10), and 21 active foraging trails on average (± sd 9.8, n=10), each at least 50 m long.

For each nest, three foraging trails were selected for assessing plant consumption by collecting each worker and the transported load in a 5 min period every 2 h over a 24-h period (**Figure 1**). Assessments were undertaken simultaneously in the three trails by three people. In site I, measurements were taken during May, July, August, and October 2016, and January and March 2017. In Site II, measurements were carried out in August, September, November, and December 2016, and January, March, April, June, and October 2017. Total daily foraging intake by the colony was assessed by multiplying the intake of one of the three measured trails over 24 h with the number of active foraging trails at the day of the measurement. This procedure was carried out for each of the three measured trails, obtaining three values of colony intake in each visit, following methods published previously (22, 25–27). Those colony intake values were averaged for the season. In total, measurements were taken at two sites, with three trails measured for each of the five colonies per site, with a total of 30 estimations of colony seasonal intake obtained.

All collected fragments were classified into three functional groups—grasses, herbs, or trees—and later weighted to obtain mass consumption. For this, the collected loads were taken to the laboratory of the Department of Ecology at the Faculty of Agronomy, Universidad Nacional de Entre Rios, Paraná, Argentina. Once in the lab, the collected material was assigned to one of the three functional groups and dried in an oven for 48 h at 60°C, after which it was weighed at a resolution of 0.1 mg. The values of seasonal consumption, as explained above, were divided into functional groups and multiplied by the number of days of each season and nest density of the site to obtain the consumption by colonies in a hectare per season or year.

Average consumption by *A. vollenweideri* colonies was compared using a two-way repeated measures ANOVA with *season* and *functional group of consumed plants* as factors. To correct for the heterogeneity of variances of the data, given that the assumption of sphericity could not be met, we applied a Greenhouse–Geisser correction factor for the degrees of freedom and reported a corrected Fc. *Post hoc* comparisons were performed using Bonferroni correction. To assess differences among functional groups inside each season, we conducted a one-way ANOVA.

### 2.2 EIL calculation

To assess the degree to which *A. vollenweideri* negatively affects cattle raising, we considered the animal unit equivalent (28, 29) for a cow (CUE), which is the yearly forage demand of one mature cow of approximately 400 kg, raising a calf up to 6 months with a daily dry-matter forage allocation of 2.5% of its weight (28, 29). The commercial goal was to sell a 160-kg calf by month six. The negative impact of ants was considered against the way ants affect the carrying capacity of the site to sustain a CUE. Carrying capacity (K) was calculated considering the typical ANPP for the areas with *A. vollenweideri* nests, multiplied by the harvesting index depending on the ANPP (30), and divided by the forage demand determined by the CUE. The K to sustain a CUE is reduced by the presence of ant nests, which compete for forage, with a concomitant reduction in weight loss of the calf. Losses due to ant presence were used to calculate the economic injury level (EIL) according to Pedigo et al. (1986), where EIL=C/VDK. The cost of control (C) of a single nest included the time needed to reach the area, locate the nests, and apply the baits, and the cost of baits ($41 USD/ha considering 1 nest/ha). The market value (V) was set depending on the local sale price of a calf (USD/kg) (31). The loss in final product weight (D) caused by a nest (kg/nest) was defined in this study (see Results). Finally, K represents the percentage of control efficiency, reported as approximately 0.9 for *A. vollenweideri* (32), under the assumption of bait acceptance, which did not occur in several cases (5). Therefore, the EIL is the number of colonies for which the costs of controlling equal the benefits of control, i.e., nest densities above this EIL justify the undertaking of control measures.

### 2.3 Landscape level density of nests

**Figure 2:**
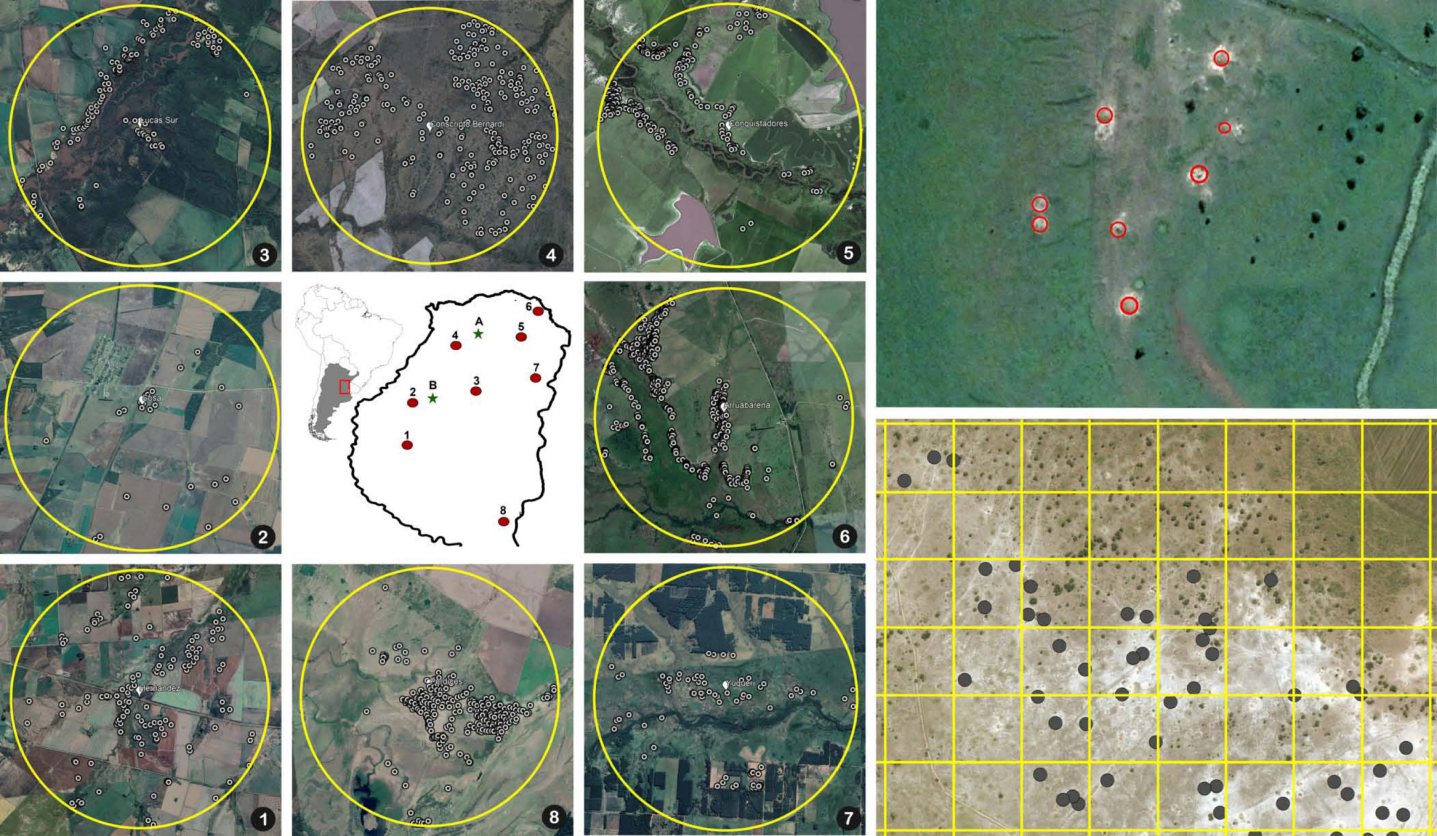
Map in the center: the numbers show the location of the eight circular areas in the province of Entre Rios, Argentina, where the nest censuses were performed. The letters show the two sites where foraging intake measurements were conducted (A, El Caraya; B, Santa Clara). Numbered pictures around the map: satellite images showing the locations of the nests in the eight areas where censuses were conducted. Upper right: detail of a field showing the position of eight nests highlighted inside red circles. Bottom right: detail of an 800 × 600 m area showing the 100 × 100 grid used for counting nest density and the marked nests inside the grid.

To determine the extent to which *A. vollenweideri* surpasses the EIL level at the landscape scale, we estimated the density of nests/ha in eight circular areas of 5 km in diameter in the province of Entre Rios, Argentina (**Figure 2**). Inside these eight areas, each nest was georeferenced using satellite images by superimposing a grid of 1 ha (100 × 100 m) and counting the nests inside each quadrant. *A. vollenweideri* nests can be observed in satellite imagery, not only because the conspicuous dome nest can reach up to 10 m in diameter, but also because they are surrounded by a halo of bare white soil lacking any vegetation, which makes it even easier to localize (8). The areas were analyzed using ArcGIS Pro 2.9.0. Only the quadrants falling entirely inside the areas were considered, making a total of 15,624 ha, in which nest density was estimated. Aerial localization of *Atta* nests has been used previously with both aerial photography (33, 34) and satellite imagery (35); the first studies of this kind were, in fact, carried out with *A. vollenweideri* in Paraguay (7, 36).

## 3 Results

### 3.1 Plant consumption by colonies

**Figure 3:**
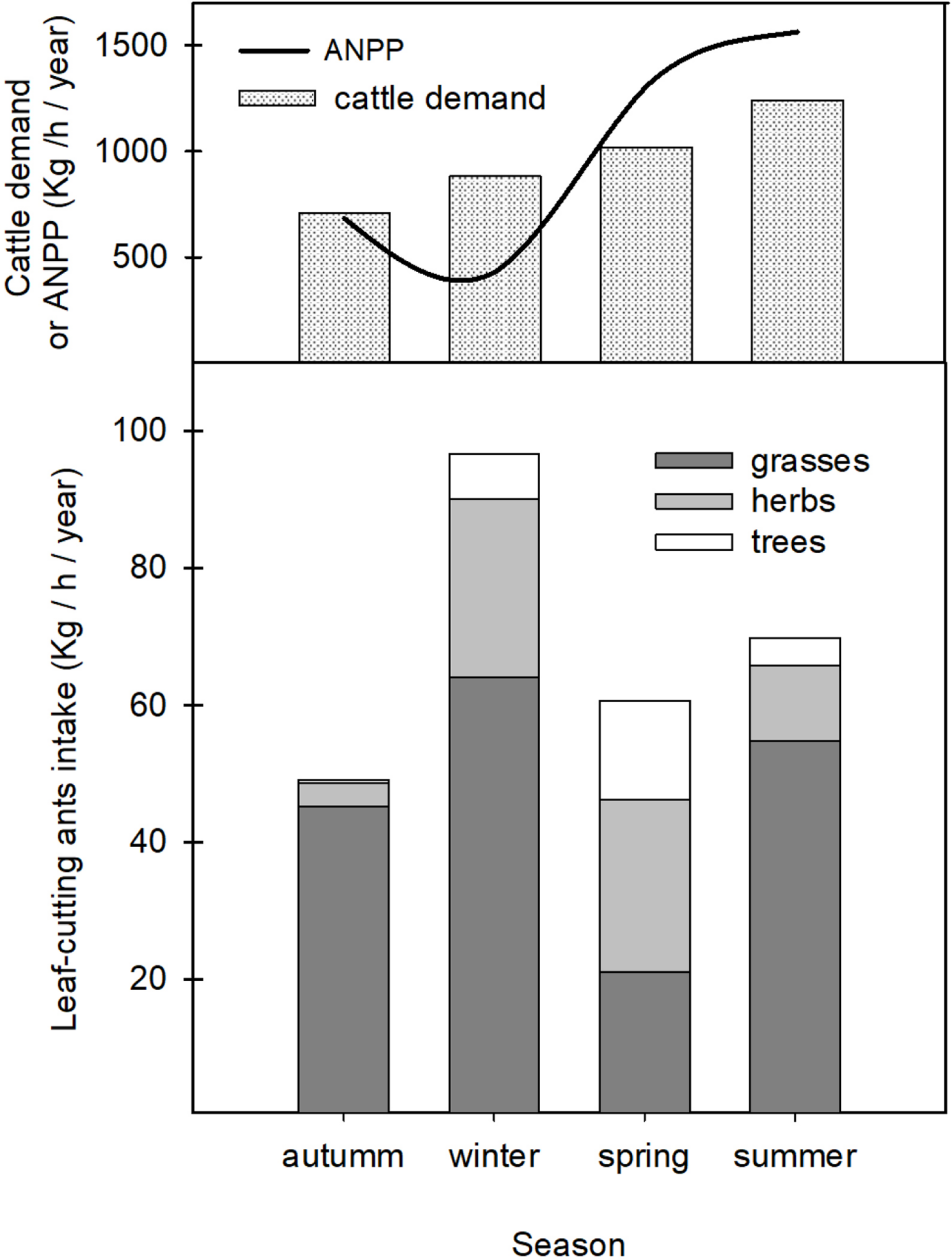
Top: estimated cattle demand as a function of season. The black continuous line is the ANPP for the region where *Atta vollenweideri* is present. See text for further explanations. Bottom: results obtained for *A. vollenweideri* consumption classified into the three functional groups (grasses, herbs, and tress) (colored stacked bars).

Results show that, in an annual average, *A. vollenweideri* consumed approximately 276 kg of dry weight/ha/year. Grasses were the functional group most consumed by ants (ca. 70%). The remaining 30% was represented by herbaceous leaves and trees (25% and 5%, respectively) (**Figure 3**, stacked bars, overall differences among colored categories, **ESM 2**).

Among seasons, there were significant differences in total plant consumption by ants. Winter consumption, with approximately 30% of the annual average, was not different from summer consumption, but higher than that of spring and autumn. Summer did not differ from the other seasons (**Figure 3**, stacked bars, overall differences among seasons) (two-way ANOVA repeated measures with *season* as the factor after a Bonferroni *post-hoc* test, F_2.16 =_ 8.76, *p*<0.0001).

When considering functional groups consumed by ants, results show that season influences the plants selected by *A. vollenweideri* workers (two-way ANOVA repeated measures with *functional group* as the factor, F_4.31 =_ 11.69, *p*<0.0001). There were significant differences among functional groups selected by workers throughout each season (**Figure 3**, stacked bars, differences inside seasons). In summer and autumn, workers consumed more grasses than herbs and trees (Summer F=58.82, *p*<0.0001; Autumn F=49.20, *p*<0.0001, Bonferroni *post-hoc* test). In winter, the season of higher consumption, there were differences among the three groups (F=36.16, *p*<0.0001, Bonferroni *post-hoc* test), with grasses being the preferred type, followed by herbs and trees to a lesser extent. In spring, there were no differences in the consumption of grasses, herbs, and trees (F=1.86, *p*=0.1652).

When comparing annual and consumption with the ANPP for the region where *A. vollenweideri* is distributed (3,846 kg of dry weight/ha/year) (37–42) (**Figure 3**), ant consumption accounts for 7.17% of total ANPP. Regarding a potential concurrence with cattle, the forage demand of a CUE is approximately 3,853 kg of dry weight/ha/year (**Figure 3**). The annual consumption by ants represents approximately 7.17% of the total CUE demand in an annual base. Although ant consumption increases during summer, overall ant consumption during spring plus summer is just 3.5% of the total cattle demand in both seasons. Even in winter, when ant demand for grasses is higher, it represents only 6% of cattle demand for that season.

### 3.2 EIL calculation

**Figure 4:**
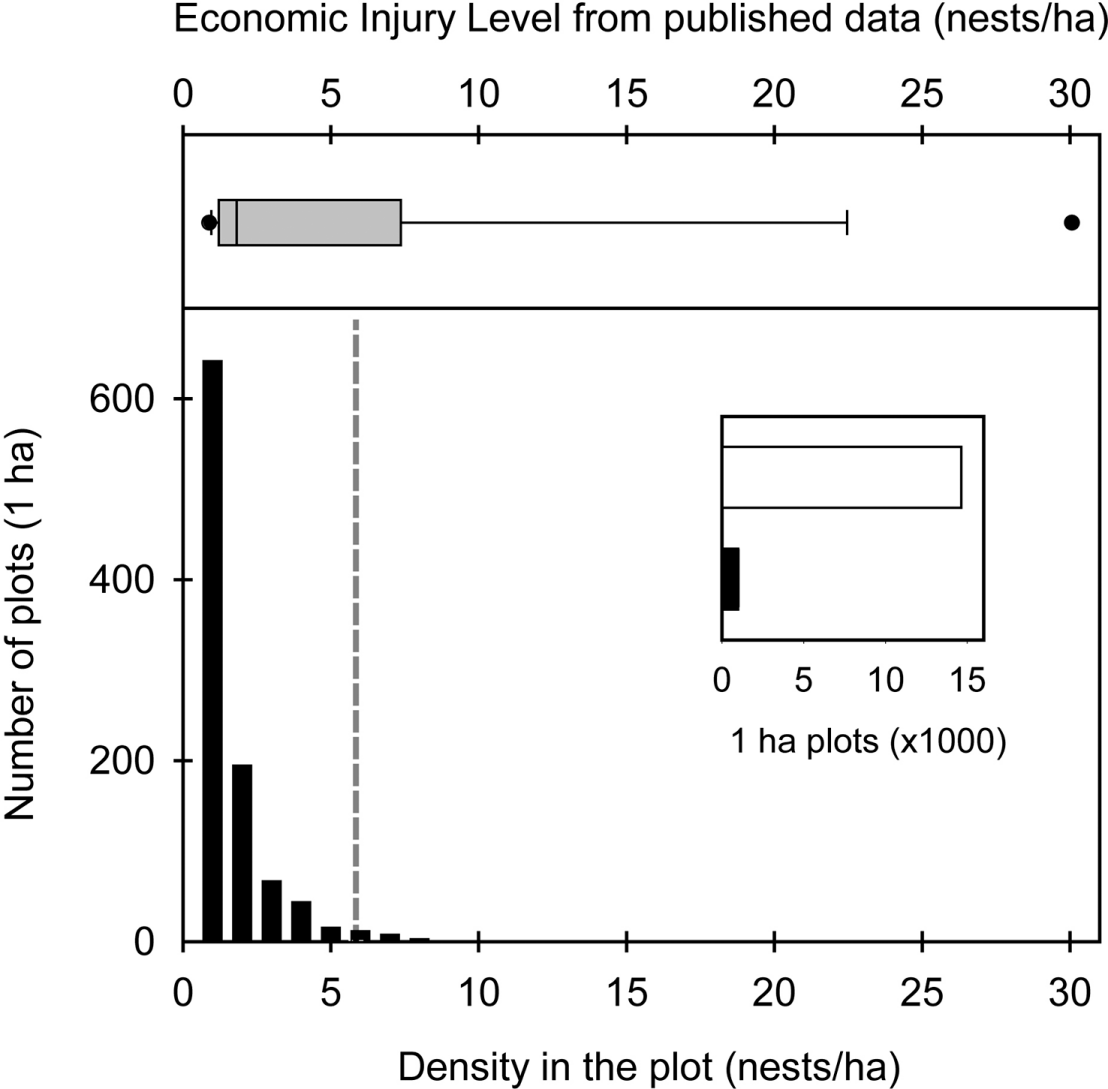
Top: average economic injury level (EIL) based on the studies detailed in **Table 1**. Bottom: number of plots with *Atta vollenweideri* as a function of its nest density as obtained in this study over an area of more than 15,000 ha. The inset shows the number of 1-ha plots without nests (white bar) vs. those with nests (black bar). The grey dashed line shows the EIL value for *A. vollenweideri* obtained in this study.

Cattle are never capable of consuming all the available forage. This, known as harvest efficiency, affects the K of the site for livestock production and, therefore, the economic injury level of ant colonies. When *A. vollenweideri* was absent (0 nest/ha), the K for livestock in the sites was approximately 0.45 CUE, given a harvesting efficiency of 0.454 (30), a cattle demand of 3,853 (dry weight kg/ha/year) for the CUE, and an ANPP of 3,846 (dry weight kg/ha/year). This means that the study sites can hold a CUE every 2.3 ha to achieve the goal of selling a 160-kg calf each year, without supplementary feed practices. From there, each *A. vollenweideri* nest reduces the carrying capacity of the site, as each colony consumes 185 dry weight kg/ha/year of grasses. On average, each nest produces a loss of 3.49 kg per calf. Thus, with a nest density of 2 nests/ha, the K decreases to 0.41 and losses rise to 6.98 kg per calf; with 5 nests/ha, the K decreases to 0.34 and calf losses rise to 17.44 kg. When considering all the costs of controlling a nest of *A. vollenweideri* (43), the EIL can be established at 5.85 nests/ha, i.e., when the benefit of controlling a population of *A. vollenweideri* surpasses its cost at densities of 6 nests/ha or above (**Figure 4**). As a standardization to allow comparisons, bibliographic data allowed us to estimate an EIL based on the consumption reported for *Atta* species (**Table 1**) by assuming the same cattle demand, harvest efficiency, and costs of control as in our study, which showed that our EIL of 5.85 falls in the range of the estimations made for several *Atta* species across six countries in the Neotropical region (**Figure 4**).

### 3.3 Landscape level density of nests

Satellite imagery censuses showed that the density at which *A. vollenweideri* starts producing economic damage (above 5.85 nests/ha) is rarely reached (**Figure 4**). Most plots showed no presence of nests (14,635 of 15,624 plots) (**ESM 2**). Among the remaining 989 plots showing evidence of nests, 964 had a density below 6 nests/ha, and only 25 equaled or surpassed this critical density. This represents 2.52% of the area holding *A. vollenweideri* populations. Moreover, the distribution of this percentage is not homogenous among sites because this EIL of 6 nests/ha was surpassed only in two of the eight surveyed sites (**Figure 2**, areas 5 and 6).

## 4 Discussion

Although grass-cutting ants have been traditionally seen as a pest of livestock production, our results show that the economic impacts of *Atta* grass-cutting ants are less important than what is commonly assumed. Although *Atta* is repeatedly cited as a grass-cutter (36, 44), our work shows that *A. vollenweideri* also cuts dicotyledonous leaves from shrubs and trees. In addition, results show that ant preferences for a specific functional group change throughout the year, with grass consumption greater in autumn but less representative in spring. These changing preferences are probably ruled by a high selectivity in response to fluctuations in palatable resources and distance to the nest, as known for other *Atta* species (45, 46). These plant preferences raise the question about competition between ants and cattle because cattle mostly consume grasses instead of shrubs and trees (28, 29). All studies to date (**Table 1**) have not differentiated the functional groups consumed by ants when comparing them with the actual dietary preferences of cattle (10–21, 47). Those studies generally applied the foraging method (9 of 13), and loads carried by ants belonging to all functional groups were pooled. In our view, this overestimated the negative impact of ants on cattle consumption. Therefore, any calculation related to economic injury levels based on published data obtained by the foraging intake method would be biased. In our case, for instance, not classifying into functional groups as we did would result in an overestimation of the percentage of non-grass loads cut by *A. vollenweideri* by approximately 33%.

During this study, we decided to classify the loads into functional groups to focus our analysis on the fraction in which the competition was more likely to occur. By doing this, we intended to overcome the extended misleading practices of previous studies that applied the foraging method. Nevertheless, and although the exclusion method for estimating ant consumption is less used than the foraging method, we believe it also has some methodological issues worth mentioning. The main difficulty of the exclusion method is that the area around nests is not homogeneously cut. Colonies periodically change activity among trails (4, 48, 49). In fact, the extent of the current foraging area was less than 25% of the potential foraging area based on an assessment of the extension of the trails (50). Additionally, cages also excluded cow trampling (28, 51, 52) and forage by other small herbivores that consume grasses (53–55). Altogether, our study and those listed in **Table 1** suffer from methodological issues that explain the great variability of values reported for *Atta* consumption and the impact they would have on livestock. However, from our point of view, our work improves on previous studies by considering functional groups using foraging methods over 24 h, increasing the number of surveyed trails, colony number, and considering the impact at a landscape level.

In addition to the method for estimating ant consumption, other factors to be taken into account are the consumption of ants and cattle in relationship with the primary productivity of the habitat and season. Farmers tend to maximize the management regime, i.e., the number of cows per hectare, for the consumption of all the ANPP under a high-pressure grazing regime (41). In this case, the probability that the consumption by any other herbivore negatively affects cattle intake is greater than when practicing a lower pressure grazing regime. However, competition occurs only if both herbivores forage on the same portion of the plants. It is known that cattle mostly cut portions of plants located above 5–10 cm (28, 56). On the contrary, there is no evidence that ants also focus on the biomass above 5–10 cm or that they cannot cut below this height. There is also no evidence to date indicating that forage consumed by ants could be taken by larger herbivores when ants are not present in that area, which seems to be an open question not only for ants but also for other small-large herbivore interactions (57). In fact, leaf-cutting ant colonies of both genera (*Atta* and *Acromyrmex*) are known to sustain stable populations in modified habitats (58–62), such as those aimed at livestock production, as commonly seen in other ant species (63). By lowering the vegetation, large herbivores promote an increase in soil temperature, which would be a limiting factor for the establishment of leaf-cutting ant colonies (62, 64), at least in southern South America (65). Intensive rangeland practices do not seem to imply the displacement of leaf-cutting ants (62, 66).

The nest density recommended to control colonies (EIL) is far beyond the density at which *Atta* colonies occur. Previous studies conducted by Jonkman (7) using aerial photography to survey the density of *A. vollenweideri* nests over an area of 80,000 km^2^ in Paraguay showed that only 10% of the area contained living nests. This is in line with our results, which show that a small portion of the surveyed area had colonies. We recognize that calculating EIL based on consumption data from the bibliography has a strong bias. However, all species of *Atta* are expected to forage annual amounts within a similar range. First, most species possess nests of different shapes but similar sizes (67, 68), which results in similar colony sizes and consumption (69, 70). Second, fungus gardens cultivated by all species are highly conserved across species at a continental scale (3, 71) and, therefore, are expected to decay foraged material in a similar way. We believe this common EIL is an underestimation because we consider toxic baits as the standard control measure, following the established rationale for all leaf-cutting ants (72). Nevertheless, it is known that grass-cutting ants of both genera, *Atta* and *Acromyrmex*, do not always accept baits (5). Species not accepting baits should be controlled with thermal fog, which is not just more costly but also extremely harmful to the environment compared with baits (5). Although we consider this common EIL to be robust, we believe it is an underestimation. Our main goal was to obtain an index that would allow us to carry out an overall approximation for the genus, not species- or location-specific comparisons. In summary, the EIL obtained during our work falls in the range of the overall median for all studies summarized in **Table 1**, which is 5.42 nests/ha (**Figure 4**). To date, average reported nest densities for *Atta* species fall mostly below this average EIL, in both natural and modified habitats (73–75).

In recent decades, it was mostly assumed that *Atta* was a severe pest of extensive livestock production. Given our work, it could be said there is no conclusive evidence for *Atta* to be considered a severe pest. In addition, we should consider the added negative effect of controlling a species without numerical evidence supporting such a decision. Several *Atta* species have been confirmed as key engineer species (76). An irrational eradication from areas aimed at extensive livestock production would preclude extremely important ecosystem processes related to the presence of *Atta* nests (9, 77, 78). Our data, similar to the previously published works, suggest that *Atta* species, despite being conspicuous herbivores in their habitats, should not always be considered a pest of livestock production in rangelands. This differs from past and current policies concerning grass-cutting ant control in the countries where *A. vollenweideri* is widely distributed. For instance, decades ago, Argentina, Paraguay, and Uruguay promoted and made the control of *A. vollenweideri* mandatory through laws and their subsequent regulatory acts (79–81), which are still in use. Now, and based on the current evidence, new regulations are needed. These should include the concept that a species is a pest in a specific production system at a given population level, i.e., the critical economic injury level, as discussed in this study, for livestock production in rangelands. It is now time to apply the methods already developed for estimating the population density of *Atta* nests and using them as a decision criterion in rational pest management practices (1, 34, 35).

## 5 Conclusion

Results show that *A. vollenweideri* consumed approximately 276 kg dry weight/ha/year of plants, the most foraged being grasses (70%) but also cutting herbs (25%) and trees (5%). This consumption represents 7% of the pasture demanded to raise a calf according to the management regime applied by farmers. Our calculated EIL was 5.85 nests/ha, which falls in the range of previous works. Colonies were absent in 93.6% of a surveyed area of 15,000 ha, while their density was below the EIL in 6.2% of the area and surpassed the EIL in only 0.2%.

These results question the perception that *Atta* leaf-cutting ants are a pest of livestock production. Although ants consume a small percentage of cattle’s demand, evidence that ants and cattle are competing in the few cases that density surpasses the EIL is arguable. In the countries where *A. vollenweideri* is present, decision makers have promulgated several acts making its control mandatory. It is time to revisit these regulations and the pest status of *A. vollenweideri* by including the use of EIL as a control criterion.

## Data availability statement

The original contributions presented in the study are included in the article/**Supplementary Material**. Further inquiries can be directed to the corresponding author.

## Author contributions

Conceptualization: JS and MB. Funding acquisition: JS and MB. Data acquisition: JS. Analysis: JS and MB. First draft writing: MB. Writing review and editing: MB. All authors contributed to the article and approved the submitted version.

## Supplementary material

ESM 1 Video showing the foraging activity in a trail of an *Atta vollenweideri* nest. The different functional groups consumed can be observed while being carried by workers into the nest.
