# Supplementary material for: Herbivory by Atta vollenweideri: Reviewing the significance of grass-cutting ants as a pest of livestock
Source: Front Insect Sci. 2023 Apr 5;3:1101445. doi: 10.3389/finsc.2023.1101445 (PMC10926485; doi:10.3389/finsc.2023.1101445)
Supplement: Supplementary file 1 [file DataSheet_1.pdf]

Electronic Supplementary Material 2:

Fig 3) Raw data of dry weight of fragments foraged by Atta vollenweideri workers classified by functional groups and pooled per study site, nest, trail and season.

Site	Nest	Trail	Fraction	Season	Dry weight
A	1	1	grasses	Autumm	56.5766154
A	1	2	grasses	Autumm	51.504903
A	1	3	grasses	Autumm	64.73131632
A	2	1	grasses	Autumm	61.39817496
A	2	2	grasses	Autumm	48.70868364
A	2	3	grasses	Autumm	54.96180048
A	3	1	grasses	Autumm	77.7797532
A	3	2	grasses	Autumm	56.89806444
A	3	3	grasses	Autumm	75.57538032
A	4	1	grasses	Autumm	81.15215148
A	4	2	grasses	Autumm	83.1753792
A	4	3	grasses	Autumm	161.9608489
A	5	1	grasses	Autumm	64.67868036
A	5	2	grasses	Autumm	58.98889152
A	5	3	grasses	Autumm	33.86658708
B	6	1	grasses	Autumm	34.51190829
B	6	2	grasses	Autumm	25.19406107
B	6	3	grasses	Autumm	39.79233642
B	7	1	grasses	Autumm	9.386393157
B	7	2	grasses	Autumm	5.642387832
B	7	3	grasses	Autumm	44.26370796
B	8	1	grasses	Autumm	15.4622514
B	8	2	grasses	Autumm	11.53171096
B	8	3	grasses	Autumm	11.28848077
B	9	1	grasses	Autumm	12.6964814
B	9	2	grasses	Autumm	61.95042384
B	9	3	grasses	Autumm	27.61062666
B	10	1	grasses	Autumm	12.0834338
B	10	2	grasses	Autumm	7.493722344
B	10	3	grasses	Autumm	4.773540367
A	1	1	grasses	Winter	55.78144272
A	1	2	grasses	Winter	130.3435368
A	1	3	grasses	Winter	83.92636584
A	2	1	grasses	Winter	105.3514901
A	2	2	grasses	Winter	77.36113008
A	2	3	grasses	Winter	121.7774304
A	3	1	grasses	Winter	78.44940936
A	3	2	grasses	Winter	158.0693087
A	3	3	grasses	Winter	72.4739076
A	4	1	grasses	Winter	131.4524128
A	4	2	grasses	Winter	83.45281824
A	4	3	grasses	Winter	104.2977146
A	5	1	grasses	Winter	117.1708157

A	5	2	grasses	Winter	64.27502064
A	5	3	grasses	Winter	44.36436852
B	6	1	grasses	Winter	71.84916288
B	6	2	grasses	Winter	55.34406
B	6	3	grasses	Winter	47.5817184
B	7	1	grasses	Winter	11.9425212
B	7	2	grasses	Winter	14.04225792
B	7	3	grasses	Winter	10.410444
B	8	1	grasses	Winter	67.42913616
B	8	2	grasses	Winter	7.66596384
B	8	3	grasses	Winter	31.21240392
B	9	1	grasses	Winter	10.4154732
B	9	2	grasses	Winter	12.64724928
B	9	3	grasses	Winter	10.506456
B	10	1	grasses	Winter	46.80484416
B	10	2	grasses	Winter	68.67253728
B	10	3	grasses	Winter	26.48184696
A	1	1	grasses	Spring	5.78204032
A	1	2	grasses	Spring	10.54503072
A	1	3	grasses	Spring	15.46851744
A	2	1	grasses	Spring	13.16406256
A	2	2	grasses	Spring	26.05159888
A	2	3	grasses	Spring	32.43782152
A	3	1	grasses	Spring	6.77804856
A	3	2	grasses	Spring	17.55345696
A	3	3	grasses	Spring	19.19015952
A	4	1	grasses	Spring	18.7813164
A	4	2	grasses	Spring	23.653256
A	4	3	grasses	Spring	13.43166944
A	5	1	grasses	Spring	6.54639296
A	5	2	grasses	Spring	21.21521936
A	5	3	grasses	Spring	8.70656024
B	6	1	grasses	Spring	41.9631876
B	6	2	grasses	Spring	8.69374944
B	6	3	grasses	Spring	22.2647256
B	7	1	grasses	Spring	9.01241784
B	7	2	grasses	Spring	13.30753752
B	7	3	grasses	Spring	11.96044344
B	8	1	grasses	Spring	26.32941648
B	8	2	grasses	Spring	4.54648824
B	8	3	grasses	Spring	11.77143696
B	9	1	grasses	Spring	55.7386236
B	9	2	grasses	Spring	37.04572728
B	9	3	grasses	Spring	31.55283504
B	10	1	grasses	Spring	45.82305288
B	10	2	grasses	Spring	39.82660056
B	10	3	grasses	Spring	31.29040224
A	1	1	grasses	Summer	12.99562488
A	1	2	grasses	Summer	35.8751916
A	1	3	grasses	Summer	22.80562992
A	2	1	grasses	Summer	29.95919136
A	2	2	grasses	Summer	26.3901564

A	2	3	grasses	Summer	44.65571472
A	3	1	grasses	Summer	12.49567128
A	3	2	grasses	Summer	29.54056824
A	3	3	grasses	Summer	57.94919928
A	4	1	grasses	Summer	33.01242912
A	4	2	grasses	Summer	43.38752256
A	4	3	grasses	Summer	104.6684549
A	5	1	grasses	Summer	18.5299704
A	5	2	grasses	Summer	49.71686472
A	5	3	grasses	Summer	22.65388344
B	6	1	grasses	Summer	117.193756
B	6	2	grasses	Summer	101.2916999
B	6	3	grasses	Summer	94.03195824
B	7	1	grasses	Summer	47.29304232
B	7	2	grasses	Summer	106.5067974
B	7	3	grasses	Summer	64.84691628
B	8	1	grasses	Summer	56.46067956
B	8	2	grasses	Summer	84.80643948
B	8	3	grasses	Summer	74.01441636
B	9	1	grasses	Summer	75.40412148
B	9	2	grasses	Summer	37.53218808
B	9	3	grasses	Summer	27.26219592
B	10	1	grasses	Summer	91.58804136
B	10	2	grasses	Summer	72.61840188
B	10	3	grasses	Summer	46.57761576
A	1	1	herbs	Autumm	3.94910532
A	1	2	herbs	Autumm	1.122255
A	1	3	herbs	Autumm	0.11671452
A	2	1	herbs	Autumm	1.02578508
A	2	2	herbs	Autumm	0.4964328
A	2	3	herbs	Autumm	0.89921232
A	3	1	herbs	Autumm	0.06407856
A	3	2	herbs	Autumm	0.71718696
A	3	3	herbs	Autumm	0.8133048
A	4	1	herbs	Autumm	7.75931508
A	4	2	herbs	Autumm	2.84727096
A	4	3	herbs	Autumm	3.56252148
A	5	1	herbs	Autumm	1.0245528
A	5	2	herbs	Autumm	4.53637476
A	5	3	herbs	Autumm	0.29205036
B	6	1	herbs	Autumm	0.664719372
B	6	2	herbs	Autumm	0.442274692
B	6	3	herbs	Autumm	0
B	7	1	herbs	Autumm	6.193861835
B	7	2	herbs	Autumm	12.47728512
B	7	3	herbs	Autumm	0.655824669
B	8	1	herbs	Autumm	3.784617128
B	8	2	herbs	Autumm	0
B	8	3	herbs	Autumm	0
B	9	1	herbs	Autumm	3.764823196
B	9	2	herbs	Autumm	0.347391963
B	9	3	herbs	Autumm	0.639746499

B	10	1	herbs	Autumm	16.73443533
B	10	2	herbs	Autumm	13.58351976
B	10	3	herbs	Autumm	12.94225783
A	1	1	herbs	Winter	8.85498804
A	1	2	herbs	Winter	15.52285512
A	1	3	herbs	Winter	5.66795988
A	2	1	herbs	Winter	26.8927506
A	2	2	herbs	Winter	52.3930248
A	2	3	herbs	Winter	18.73928196
A	3	1	herbs	Winter	44.74426284
A	3	2	herbs	Winter	30.70454472
A	3	3	herbs	Winter	8.36242812
A	4	1	herbs	Winter	69.29304084
A	4	2	herbs	Winter	73.51307172
A	4	3	herbs	Winter	23.57738928
A	5	1	herbs	Winter	22.23438012
A	5	2	herbs	Winter	10.82927664
A	5	3	herbs	Winter	15.76983924
B	6	1	herbs	Winter	17.22327264
B	6	2	herbs	Winter	25.22765592
B	6	3	herbs	Winter	22.42849464
B	7	1	herbs	Winter	24.65414424
B	7	2	herbs	Winter	32.15377872
B	7	3	herbs	Winter	16.12919304
B	8	1	herbs	Winter	28.48785768
B	8	2	herbs	Winter	13.69752912
B	8	3	herbs	Winter	10.12716288
B	9	1	herbs	Winter	34.18603272
B	9	2	herbs	Winter	17.57229912
B	9	3	herbs	Winter	19.69498728
B	10	1	herbs	Winter	29.88323208
B	10	2	herbs	Winter	38.73178944
B	10	3	herbs	Winter	23.19823656
A	1	1	herbs	Spring	4.47326768
A	1	2	herbs	Spring	10.3640616
A	1	3	herbs	Spring	4.66808528
A	2	1	herbs	Spring	16.01765792
A	2	2	herbs	Spring	43.3119688
A	2	3	herbs	Spring	36.31510904
A	3	1	herbs	Spring	8.46873672
A	3	2	herbs	Spring	17.11546944
A	3	3	herbs	Spring	4.52686208
A	4	1	herbs	Spring	5.36525584
A	4	2	herbs	Spring	21.24140368
A	4	3	herbs	Spring	13.25890248
A	5	1	herbs	Spring	5.66936168
A	5	2	herbs	Spring	6.22189256
A	5	3	herbs	Spring	5.58517544
B	6	1	herbs	Spring	59.37528384
B	6	2	herbs	Spring	33.83133696
B	6	3	herbs	Spring	54.3949128
B	7	1	herbs	Spring	20.62191456

B	7	2	herbs	Spring	37.49762376
B	7	3	herbs	Spring	17.56178352
B	8	1	herbs	Spring	53.40516624
B	8	2	herbs	Spring	35.97167304
B	8	3	herbs	Spring	34.88198256
B	9	1	herbs	Spring	9.00080496
B	9	2	herbs	Spring	14.1315948
B	9	3	herbs	Spring	61.88266008
B	10	1	herbs	Spring	37.72640664
B	10	2	herbs	Spring	61.86711528
B	10	3	herbs	Spring	20.0514204
A	1	1	herbs	Summer	1.0069488
A	1	2	herbs	Summer	0
A	1	3	herbs	Summer	0.61473168
A	2	1	herbs	Summer	1.23896952
A	2	2	herbs	Summer	0
A	2	3	herbs	Summer	2.90466
A	3	1	herbs	Summer	0
A	3	2	herbs	Summer	0.39609
A	3	3	herbs	Summer	0.40982112
A	4	1	herbs	Summer	5.16219696
A	4	2	herbs	Summer	4.418604
A	4	3	herbs	Summer	3.5208
A	5	1	herbs	Summer	0.36405072
A	5	2	herbs	Summer	2.80889424
A	5	3	herbs	Summer	3.41658432
B	6	1	herbs	Summer	37.86000048
B	6	2	herbs	Summer	28.66067928
B	6	3	herbs	Summer	24.8485914
B	7	1	herbs	Summer	23.66846676
B	7	2	herbs	Summer	12.23439768
B	7	3	herbs	Summer	44.22628188
B	8	1	herbs	Summer	17.39092788
B	8	2	herbs	Summer	12.55499546
B	8	3	herbs	Summer	33.71996304
B	9	1	herbs	Summer	2.1829014
B	9	2	herbs	Summer	16.342614
B	9	3	herbs	Summer	5.328666
B	10	1	herbs	Summer	10.11253248
B	10	2	herbs	Summer	23.59933812
B	10	3	herbs	Summer	10.89585324
A	1	1	trees	Autumm	0
A	1	2	trees	Autumm	0
A	1	3	trees	Autumm	0
A	2	1	trees	Autumm	0
A	2	2	trees	Autumm	0
A	2	3	trees	Autumm	0
A	3	1	trees	Autumm	0
A	3	2	trees	Autumm	0
A	3	3	trees	Autumm	0
A	4	1	trees	Autumm	0
A	4	2	trees	Autumm	0

A	4	3	trees	Autumm	0
A	5	1	trees	Autumm	0
A	5	2	trees	Autumm	0
A	5	3	trees	Autumm	0
B	6	1	trees	Autumm	4.374944805
B	6	2	trees	Autumm	0.158788133
B	6	3	trees	Autumm	5.598883411
B	7	1	trees	Autumm	0
B	7	2	trees	Autumm	0.306386691
B	7	3	trees	Autumm	0.272502697
B	8	1	trees	Autumm	0.03218695
B	8	2	trees	Autumm	0
B	8	3	trees	Autumm	0.049692081
B	9	1	trees	Autumm	0.466192575
B	9	2	trees	Autumm	0
B	9	3	trees	Autumm	3.840606345
B	10	1	trees	Autumm	0
B	10	2	trees	Autumm	0.210923377
B	10	3	trees	Autumm	0.582589318
A	1	1	trees	Winter	0.85520232
A	1	2	trees	Winter	0
A	1	3	trees	Winter	0
A	2	1	trees	Winter	0
A	2	2	trees	Winter	0
A	2	3	trees	Winter	24.46463088
A	3	1	trees	Winter	3.65934348
A	3	2	trees	Winter	10.50765156
A	3	3	trees	Winter	0
A	4	1	trees	Winter	9.2852298
A	4	2	trees	Winter	12.82909104
A	4	3	trees	Winter	7.8144156
A	5	1	trees	Winter	1.41518556
A	5	2	trees	Winter	3.01098816
A	5	3	trees	Winter	0.5034744
B	6	1	trees	Winter	18.04321512
B	6	2	trees	Winter	9.22867344
B	6	3	trees	Winter	2.28590856
B	7	1	trees	Winter	17.86198104
B	7	2	trees	Winter	11.13254568
B	7	3	trees	Winter	38.68424064
B	8	1	trees	Winter	0
B	8	2	trees	Winter	5.66461656
B	8	3	trees	Winter	0.93287088
B	9	1	trees	Winter	0.70024752
B	9	2	trees	Winter	4.60071216
B	9	3	trees	Winter	2.25152712
B	10	1	trees	Winter	1.12398048
B	10	2	trees	Winter	3.80417832
B	10	3	trees	Winter	4.71089736
A	1	1	trees	Spring	0
A	1	2	trees	Spring	0
A	1	3	trees	Spring	0

A	2	1	trees	Spring	0
A	2	2	trees	Spring	0
A	2	3	trees	Spring	0
A	3	1	trees	Spring	21.83615808
A	3	2	trees	Spring	0
A	3	3	trees	Spring	0
A	4	1	trees	Spring	0.27365744
A	4	2	trees	Spring	0
A	4	3	trees	Spring	0
A	5	1	trees	Spring	0
A	5	2	trees	Spring	0
A	5	3	trees	Spring	21.37017368
B	6	1	trees	Spring	12.1756932
B	6	2	trees	Spring	94.90713048
B	6	3	trees	Spring	4.0087296
B	7	1	trees	Spring	5.64559704
B	7	2	trees	Spring	12.18694032
B	7	3	trees	Spring	6.96900816
B	8	1	trees	Spring	8.35871328
B	8	2	trees	Spring	5.00332248
B	8	3	trees	Spring	7.45409736
B	9	1	trees	Spring	135.8278106
B	9	2	trees	Spring	32.40560448
B	9	3	trees	Spring	31.8636396
B	10	1	trees	Spring	11.40677424
B	10	2	trees	Spring	18.464022
B	10	3	trees	Spring	5.04629928
A	1	1	trees	Summer	0.3943296
A	1	2	trees	Summer	0
A	1	3	trees	Summer	0
A	2	1	trees	Summer	0.2006856
A	2	2	trees	Summer	0
A	2	3	trees	Summer	0
A	3	1	trees	Summer	0
A	3	2	trees	Summer	0.5703696
A	3	3	trees	Summer	0
A	4	1	trees	Summer	5.54666832
A	4	2	trees	Summer	0
A	4	3	trees	Summer	0.387288
A	5	1	trees	Summer	2.4786432
A	5	2	trees	Summer	1.2358008
A	5	3	trees	Summer	0
B	6	1	trees	Summer	6.0062364
B	6	2	trees	Summer	10.90778616
B	6	3	trees	Summer	1.46651472
B	7	1	trees	Summer	6.90792624
B	7	2	trees	Summer	3.06799488
B	7	3	trees	Summer	2.41566192
B	8	1	trees	Summer	9.71614008
B	8	2	trees	Summer	6.53265648
B	8	3	trees	Summer	4.52175372
B	9	1	trees	Summer	34.59806136

B	9	2	trees	Summer	3.439287
B	9	3	trees	Summer	12.7277622
B	10	1	trees	Summer	5.60271168
B	10	2	trees	Summer	3.89287512
B	10	3	trees	Summer	0

Fig 4) Number of plots with A. vollenweideri as a function of its nest density

number of plots	nest/ha
14635	0
652	1
196	2
60	3
42	4
15	5
11	6
8	7
3	8
1	9
1	10
